# Phenotypes, mechanisms, and therapeutic strategies of natural killer cell immunosenescence

**DOI:** 10.1186/s12979-025-00534-8

**Published:** 2025-10-21

**Authors:** Zixuan Guo, Fengtian Wu, Yao Chen, Jia Xu, Zhi Chen

**Affiliations:** 1 State Key Laboratory for Diagnosis and Treatment of Infectious Diseases, National Clinical Research Center for Infectious Diseases, China-Singapore Belt and Road Joint Laboratory on Infection Research and Drug Development, National Medical Center for Infectious Diseases, Collaborative Innovation Center for Diagnosis and Treatment of Infectious Diseases, The First Affiliated Hospital, Zhejiang University School of Medicine, 79 Qingchun Road, Hangzhou, Zhejiang Province 310003 China; 2 Yuhang Institute for Collaborative Innovation and Translational Research in Life Sciences and Technology, Hangzhou, China

**Keywords:** NK cells, Immunosenescence, Cytokine dysregulation, Adoptive NK cell therapy

## Abstract

**Background:**

Natural killer (NK) cells serve as pivotal effector cells within the innate immune system, playing an indispensable role against infections and tumors. Individuals with diminished NK cell activity or NK cell deficiency are at a higher risk of developing cancers and experiencing severe viral infections. With global demographic shifts toward aging populations, elucidating the mechanisms of immunosenescence becomes increasingly critical for developing targeted therapeutic interventions against age-related disorders.

**Main body of the abstract:**

This review provides a comprehensive summary of the phenotypic characteristics, functional changes, and mechanisms of NK cells in aging and diseases. By synthesizing current research, it highlights key pathways contributing to NK cell immunosenescence in the elderly and explores potential strategies to preserve or restore their cytotoxic and immunoregulatory functions.

**Short conclusion:**

The review provides novel insights into NK cell immunosenescence and proposes innovative approaches to enhance NK cell activity in aging individuals, offering potential therapeutic avenues for mitigating age-related immune decline.

## Background

Natural killer (NK) cells are integral components of the innate immune system and also play a crucial role in adaptive immunity. Within the circulating lymphocyte population, NK cells account for approximately 5%−20% and can be classified into two principal subsets based on differential surface expression of CD56 and CD16: CD56^bright^CD16^−^ NK cells and CD56^dim^CD16^+^ NK cells [1]. The CD56^dim^CD16^+^ NK cells, which make up about 90% of NK cells in the peripheral blood, are considered mature NK cells (mNK cells) with high cytotoxic activity [2]. Conversely, the less abundant subset, comprising only about 10%, known as CD56^bright^CD16^−^ NK cells or immature NK cells (iNK cells), produces abundant cytokines and chemokines upon stimulation with IL-2, IL-12, or IL-18 [1]. It is worth noting that CD56^bright^CD16^−^ NK cells have the ability to differentiate into mature CD56^dim^CD16^+^ NK cells. Additionally, another distinct subset of NK cells called CD56^−^CD16^+^ has been identified in chronic human immunodeficiency virus (HIV) infection [3]. Unlike conventional CD56^dim^ NK cells, these particular cells exhibit impaired secretion of interferon-gamma (IFN-γ) along with direct target cell elimination and antibody-dependent cell-mediated cytotoxicity (ADCC) [3–5].

Immunosenescence arises from various sources of damage signals, such as oxidative stress, mitochondrial dysfunction, epigenetic modifications, and persistent DNA damage [6]. This phenomenon is characterized by the destruction and remodeling of immune organ structure, along with the dysfunction of innate and adaptive immunity [6, 7]. Consequently, individuals become more vulnerable to infections, malignancies, and autoimmune disorders [6, 8, 9]. Multiple metabolic changes occur during immunosenescence, including altered glycolysis, mitochondrial dysfunction, and increased production of reactive oxygen species (ROS) [6, 10, 11]. Identifying immunosenescence-associated features is essential to explore its impact and clinical significance. In this article, we describe the characteristics of NK cell immunosenescence, its specific mechanisms, and related diseases.

## Immunosenescence of NK cell subsets

Accumulating evidence indicates that NK cell subsets undergo significant phenotypic alterations and functional decline during immunosenescence (Table 1).

**Table 1:** Immunosenescence of NK cell subsets

Subset	Change	Reason	Characteristics	Impact
CD56^dim^CD16^+^NK cell	↑	May be attributed to the long-term accumulation of NK cells	1. A marker of replicative senescence and impaired proliferation increase: CD57↑ 2.Activating receptors decrease: NKp30↓, NKp46↓, CD69↓, CD94/NKG2A↓, CD161↓, DNAM-1↓ 3. Inhibitory receptors increase: KLRG1↑ 4.Perforin and granzyme A↓	Affect the activation and cytotoxicity of NK cells
CD56^bright^CD16^−^NK cell	↓	1. Age-related alterations in the number and functionality of hematopoietic stem cells 2. Impaired generation of new NK cells	The ability to produce cytokines decrease: IFN-γ↓, MIP-1α↓, IL-8↓	poor immune regulation, poor resolution of inflammation, and poor induction of adaptive immunity
CD56^−^CD16^+^ NK cell	↑	Persistent viral infection, such as chronic HIV, HCV, and EBV infection.	1. CD57^low^KIR^low^ phenotype 2. Low replication 3. Toxicity↓ 4. Cytokine release↓ 5. Inhibitory receptors↑ 6. Activating receptors decrease: NCRs↓, NKG2D↓ 7. Transcription factors T-bet↓	Be considered dysfunctional cells
Adaptive NK cell	-	Acute or chronic viral infections	1. Be long-lived and can remain stable in healthy donors from 6 months to 4 years 2. It is capable of memory recall and exhibits a degree of antigen specificity 3. ADCC activity increases and more IFN-γ and TNF-α secreted upon activation	-

↑ Increase, ↓ Decrease, -: No data

### CD56^dim^CD16^+^ NK cells

Current research data show that NK cell numbers and proportions in elderly populations mostly remain stable or increase, with only a few studies reporting a decline (Table 2) [12–18]. These variations may arise from differences in age ranges, gender distribution, ethnic backgrounds, health status, and methodologies used to assess NK cells among study populations. More research is still needed to draw definitive conclusions. Age-dependent alterations in NK cell subpopulations reveal a consistent decline in CD56^bright^ NK cells accompanied by expansion of CD56^dim^ subsets [15, 17, 19]. The surface marker CD57 has been identified as a marker of cellular senescence in NK cells, correlating with both replicative senescence and impaired proliferative capacity [20–22]. A cross-sectional study conducted by Le Garff-Tavernier and Segerstrom demonstrated a higher proportion of CD56^dim^CD57^+^ NK cells among the elderly, a finding that was further confirmed in a longitudinal study by Rebecca G. Reed et al. [15, 23, 24]. It has been demonstrated that CD57^+^ NK cells exhibit reduced proliferative capacity in response to cytokines or target cells compared to CD57^−^ NK cells [25]. Due to the high prevalence of cytomegalovirus (CMV) infection among older individuals, it is challenging to discern the specific effects of aging and CMV on NK cells. Recent studies that stratified donors based on CMV serology have revealed that CD57 upregulation on CD56^dim^ NK cells is associated with CMV seropositivity rather than senescence [26]. These studies also observed concomitant downregulation of various NK cell surface markers, including NKp30 and CD161 [27, 28]. Furthermore, the expression pattern and migratory capacity of perforin in CD56^dim^ NK cells are speculated to decline with aging, thereby directly impacting NK cell-mediated lysis of senescent cells [29].

**Table 2:** NK cell counts and proportions in elderly populations: variations across studies

Aging cohort characteristics [*N*, age, sex]	Country	Results (Young vs. Aged)				References
*N* = 21 (9 M, 12 F); ≥ 60 years	Brazil	Total NK	Percentage (% lymphocytes)	8.93 vs. 14.85 (*p* < 0.001)	↑	[12]
			Count (cells/mm^3^)	141 vs. 293 (*p* < 0.001)	↑	
		CD56^dim^ NK	Percentage (% NK cells)	94.52 vs. 97.45 (*p* = 0.001)	↑	
			Count (cells/mm^3^)	136 vs. 279 (*p* < 0.001)	↑	
		CD56^bright^ NK	Percentage (% NK cells)	5.48 vs. 2.59 (*p* = 0.001)	↓	
			Count (cells/mm^3^)	9 vs. 7 (*p* = 0.375)	Stable	
*N* = 21; > 60 years	United Kingdom	CD3^−^CD56^+^ NK	Percentage (% lymphocytes)	10.60 ± 0.9 vs. 17.21 ± 1.2 (*p* = 0.0001)	↑	[13]
		CD56^dim^ NK	Percentage (% lymphocytes)	10.40 ± 0.9 vs. 16.75 ± 1.2 (*p* < 0.0001)	↑	
		CD56^bright^ NK	Percentage (% lymphocytes)	0.56 ± 0.1 vs. 0.40 ± 0.04 (*p* = 0.03)	↓	
*N* = 14; 77–89 years (mean 81 ± 3)	Spain	CD3^−^CD56^+^ NK	Percentage (% lymphocytes)	14 ± 3 vs. 32 ± 10 (*p* < 0.01)	↑	[14]
			Count (cells/µL)	291 ± 87 vs. 407 ± 176 (*p* < 0.05)	↑	
		CD56^dim^ NK	Percentage (% NK cells)	92 ± 5 vs. 97 ± 4 (*p* < 0.05)	↑	
			Count (cells/µL)	280 ± 86 vs. 392 ± 148 (*p* < 0.01)	↑	
		CD56^bright^ NK	Percentage (% NK cells)	6 ± 3 vs. 3 ± 2 (NS)	Stable	
			Count (cells/µL)	25 ± 18 vs. 18 ± 14 (*p* = 0.375)	Stable	
*N* = 30; > 80 years (mean 87.1 ± 4.9)	France	CD3^−^CD56^+^ NK	Percentage (% lymphocytes)	-	↑	[15]
*N* = 41 (33 M, 8 F); 65–80 years (mean 71.6)	China	CD16^+^CD56^+^ NK	-	-	Stable	[16]
*N* = 67; > 60 years (mean 79)	United Kingdom	Total NK	Percentage (% lymphocytes)	9.25 ± 6.1 vs.11.22 ± 9.1 (NS)	Stable	[17]
			Count (cells/µL)	194.9 ± 140.4 vs.205.8 ± 184.0 (NS)	Stable	
		CD56^dim^ NK	Percentage (% lymphocytes)	8.49 ± 5.8 vs. 11.7 ± 9.3 (NS)	Stable	
			Count (cells/µL)	179.3 ± 135.3 vs. 197.7 ± 180.3 (NS)	Stable	
		CD56^bright^ NK	Percentage (% lymphocytes)	0.76 ± 0.6 vs. 0.5 ± 0.5 (*p* = 0.012)	↓	
			Count (cells/µL)	15.64 ± 12.8 vs. 8.13 ± 7.9 (*p* = 0.0004)	↓	
*N* = 17; Mean age 73.5 ± 1.6 years	China	Total NK	Percentage (%)	-	↓	[18]
			Count	-	↓	

*M* Male, *F *Female, ↑ Increase, ↓ Decrease, -: No data

### CD56^bright^CD16^−^ NK cells

CD56^bright^CD16^−^ NK cells exhibit both quantitative reduction and progressive functional decline with age, collectively contributing to deteriorated immunoregulatory capacity [30]. Decreased levels of CD56^bright^ NK cells in the elderly could be due to age-associated changes in both the quantity and functionality of hematopoietic stem cells, alongside a diminished capacity for generating new NK cells [20, 31]. A study discovered that senescent CD56^bright^ NK cells exhibited markedly reduced cytokine production levels (such as IFN-γ, MIP-1α, IL-8) compared to younger CD56^bright^ NK cells [29].

### CD56^−^CD16^+^ NK cells

The characteristics of CD56^−^CD16^+^ NK cells, which are considered dysfunctional cells, include low replication, decreased cytotoxicity, decreased cytokine secretion, increased expression of inhibitory NK receptors, and decreased expression of natural cytotoxicity receptors (NCRs) and NKG2D [3, 4, 32, 33]. CD56^−^CD16^+^ NK cells represent a mature population and expand in the elderly [26]. Compared with CD56^dim^NK cells, CD56^−^CD16^+^ NK cells have CD57^low^KIR^low^ phenotype accompanied by reduced T-bet expression and elongated telomeres [3].

### Adaptive NK cells

Adaptive NK cells are induced by acute or chronic viral infections such as CMV and HIV [34]. These specialized cells exhibit remarkable longevity, remaining stable in healthy individuals for periods ranging from 6 months to 4 years [24, 35, 36]. They also demonstrate a degree of antigen-specific immunological memory [37]. Unlike conventional NK (cNK) cells, adaptive NK cells typically show elevated NKG2C expression [36]. Upon activation, adaptive NK cells display enhanced antibody-dependent cellular cytotoxicity (ADCC) and produce greater quantities of IFN-γ and tumor necrosis factor-alpha (TNF-α) [38]. Additionally, a memory-like proinflammatory CD52^+^NKG2C^+^CD94^+^ NK subset that accumulates with aging was discovered by Guo et al. using single-cell transcriptome sequencing [39]. This subset exhibits proinflammatory features and a type I interferon-responsive state, correlating with disease severity in coronavirus disease 2019 (COVID-19) [39].

### Immunosenescence of NK cells in mice

Notably, the changes in NK cell subsets in aged mouse models are distinct from those observed in humans. Several studies indicate that aged mice exhibit a decline in total NK cell numbers [40, 41]. While mature NK cells, commonly identified as CD11b^low^CD27^high^KLRG1^+^ subset, decrease in aged mice, which results in a relative accumulation of immature CD11b^−^CD27^+^KLRG1^−^ NK cells [40, 42]. However, the frequency of mature NK cells in bone marrow remains unchanged in aged mice, suggesting that the observed reduction in circulating mature NK cells likely results from diminished maturation efficiency and impaired egress of mature NK cells from the bone marrow [40]. Further research is required to determine whether complex factors like CMV infection account for the differences in NK cell alterations between humans and mice.

## Mechanisms of NK cell immunosenescence

### Genetic basis of NK cell immunosenescence

#### Telomere shortening

Among the factors involved in the regulation of NK cell lifespan, telomere length plays a pivotal role. Telomere-dependent senescence occurs after clonal expansion of lymphocytes and loss of telomeres, which results from cell division stimulated by persistent antigens [43–45]. Telomere shortening is facilitated by cell differentiation, and as a result, mature CD56^dim^ NK cells have shorter telomeres than immature CD56^bright^ NK cells [46]. Research indicates that NK cells undergo age-dependent telomere attrition and progressive loss of telomerase activity [47]. The observed telomere shortening in senescent NK cells may result from stem cell telomere depletion, proliferation induced by immune homeostasis or viral infection, or a combination of these factors [20]. Further studies are necessary to elucidate the underlying mechanisms.

**Fig. 1:**
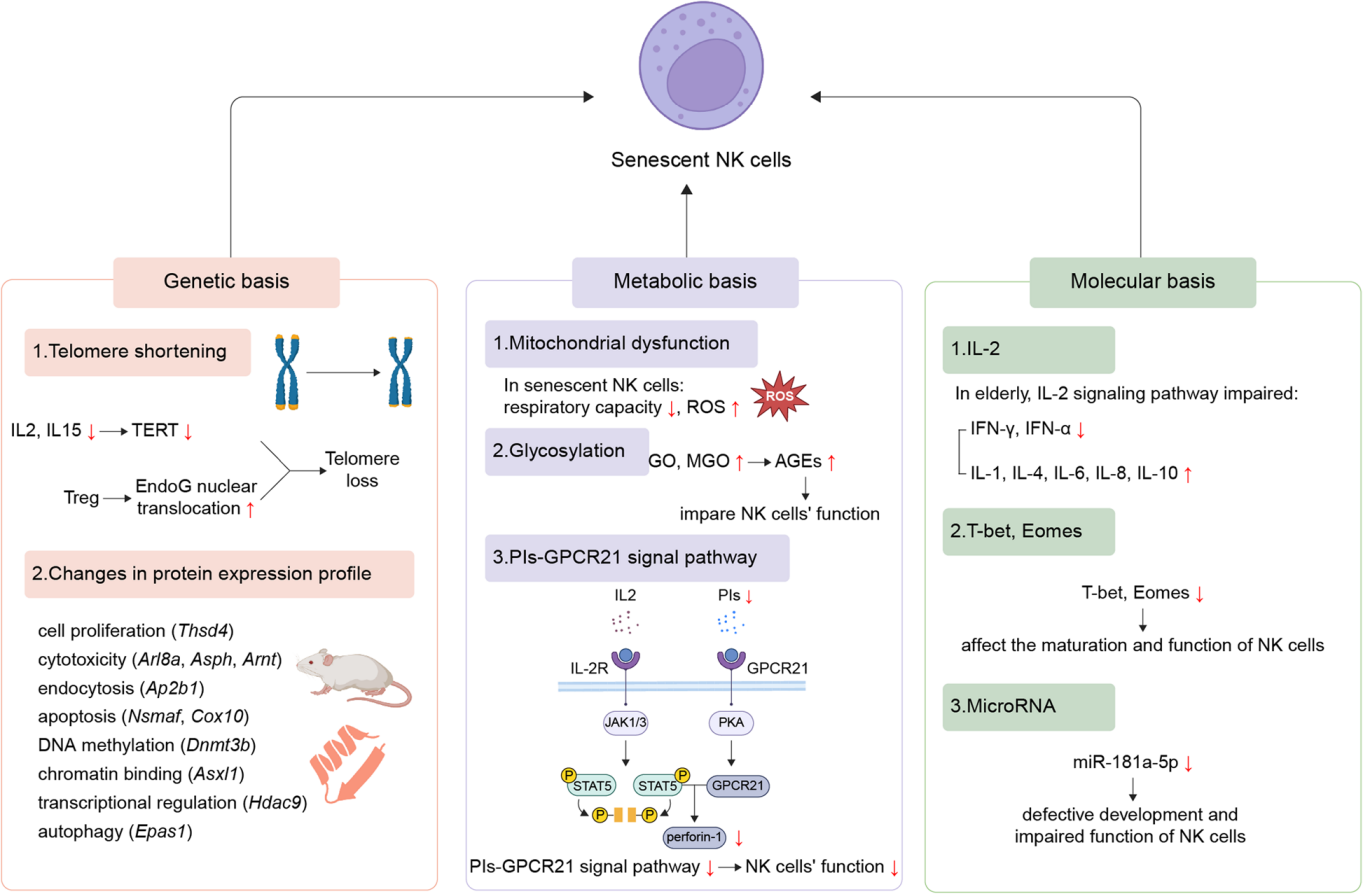
Mechanisms of NK cell immunosenescence. NK cell immunosenescence is driven by a combination of genetic, metabolic, and molecular mechanisms. EndoG: endonuclease G; GO: Glyoxal; GPCR21: G protein-coupled receptor 21; JAK1/3: Janus kinase 1/3; MGO: Methylglyoxal; NK: Natural killer; Pls: Plasmalogens; PKA: Protein kinase A; ROS: Reactive oxygen species; STAT5: Signal transducer and activator of transcription 5; TERT: Telomerase reverse transcriptase; Treg: Regulatory T cells. Created in https://BioRender.com

It has been demonstrated that IL-2 and IL-15 can enhance telomerase activity and upregulate the level of telomerase reverse transcriptase (TERT) mRNA, which in turn prevents telomere loss in NK cells [48, 49]. However, the telomere length in NK cells is influenced by the reduced availability of IL-2 and IL-15 in the aging microenvironment, as these cytokines play a critical role in NK cell homeostasis and telomere maintenance [12, 50, 51]. Furthermore, telomerase activity is inhibited by the relocation of apoptotic endonuclease G (EndoG) to the nucleus. Regulatory T (Treg) cells can regulate EndoG nuclear translocation, leading to telomere loss and cell senescence [43] (Fig. 1). Telomere shortening activates the DNA damage response (DDR) and p53-dependent apoptotic pathway, thereby inducing programmed cell death. Complete telomere loss may lead to chromosomal end-to-end fusions or breakage, resulting in genomic instability that ultimately culminates in cell necrosis or mitotic catastrophe [52].

#### Changes in protein expression profile

Using in-silico mapping techniques, S.A. Bumgardner et al. identified potential genes associated with the phenotype of senescent NK cells in mice [53]. Many of them are protein-coding genes, which were categorized based on the protein functions to screen for genes that are either directly or indirectly related to the activity of NK cells. Such protein functions include cell proliferation (*Thsd4*), cytotoxicity (*Arl8a*, *Asph*, *Arnt*), endocytosis (*Ap2b1*), apoptosis (*Nsmaf*, *Cox10*), DNA methylation (*Dnmt3b*), chromatin binding (*Asxl1*), transcriptional regulation (*Hdac9*), and autophagy (*Epas1*) [53]. These selected genes require further verification. This study reveals the genetic basis of the NK cell senescence phenotype for the first time and offers insights for further research on the mechanisms of NK cell senescence.

### Metabolic basis of NK cell immunosenescence

It has been demonstrated that cell metabolism is crucial for the generation of immune cell phenotypes and for achieving optimal immune cell function.

#### Mitochondrial dysfunction

A characteristic of NK cell senescence may be impaired cellular signaling and mitochondrial function [6, 54]. The respiratory capacity of activated NK cells is decreased, and the production of ROS is increased in the elderly, which affects the function of NK cells [6]. The researchers found that stimulation with high levels of IL-2 increased neither mitochondrial mass nor mitochondrial membrane potential in senescent NK cells, unlike in younger NK cells [54]. Furthermore, in NK cells from senior donors, IL-2 does not increase the expression of PPAR-gamma coactivator 1-alpha (PGC-1α), which can enhance mitochondrial function and metabolism and provide protection against various aging-related diseases [54]. Mitochondrial dysfunction markedly impairs cell viability and compromises antitumor activity [55].

#### Glycosylation

Glycation is a defining characteristic of molecular senescence, specifically manifested by the production of advanced glycation end products (AGEs) [56]. Two highly potent dicarbonyl compounds that generate AGEs are glyoxal (GO) and methylglyoxal (MGO), and their levels in plasma are elevated in aging and age-related diseases [57]. In vitro studies have shown that both GO and MGO interfere with the function of NK cells [57]. Furthermore, the glycosylation of tumor cells can disrupt the cytotoxic function of NK cells against tumor cells [57, 58]. Therefore, glycation exerts significant negative effects on NK cell function.

#### PIs-GPCR21 signal pathway

Plasmalogens (Pls) are a particular type of phospholipid which are reduced in the elderly [59]. NK cells specifically express G protein-coupled receptor 21 (GPCR21) on their surface, which can recognize and bind to Pls [60]. The Pls-GPCR21 signaling cascade activates signal transducer and activator of transcription 5 (STAT5), thereby inducing the expression of perforin-1 [60, 61]. However, when Pls levels decline with age, the Pls-GPCR21 signaling cascade is downregulated, leading to restricted perforin-1 expression and impaired NK cell function. Thus, the age-dependent reduction of this signaling cascade may be one of the contributing factors to NK cell immunosenescence [60].

### Molecular basis of NK cell immunosenescence

#### Cytokines

A variety of cytokines are essential for NK cell development, differentiation, and function. IL-2 is a crucial cytokine that links adaptive immunity with NK cells, serving to boost cytokine release, promote proliferation, and improve killing capacity [62]. Previous studies have reported age-related impairment of the IL-2 signaling pathway in NK cells [14]. It indicates that in the elderly, IL-2 rarely promotes the development of NK cells and generates lower levels of IFN-γ and IFN-α while raising levels of IL-1, IL-4, IL-6, IL-8, and IL-10 [63, 64]. IL-2 has been used as an immunotherapeutic agent to promote the antitumor activity of NK cells and is currently used in the treatment of metastatic renal cell carcinoma (MRCC) and melanoma [62]. Additionally, IL-12, IL-15, and IL-18 also play an important role in the immunosenescence of NK cells [49, 65–67].

#### Transcription factors T-bet and Eomes

The transcription factor T-bet (encoded by *Tbx21*) is a tyrosine- and serine-phosphorylated protein exhibiting hematopoietic lineage-restricted expression [68]. Eomes (Eomesodermin) is another transcription factor critical for the development, differentiation, and function of immune cells, particularly in regulating the cytotoxic activity of CD8^+^ T cells and NK cells [68, 69]. Both of them are downregulated in the process of NK cell senescence and are associated with the impaired cytotoxicity of NK cells [42]. In aged mice, T-bet and Eomes expression in the bone marrow is inversely correlated with poor NK cell maturation [42, 70]. Hesham M et al. constructed bone marrow chimeras from young and old individuals respectively, and examined how NK cells developed under the same conditions in the two groups. They discovered that one factor contributing to NK cell maturation and functional impairment was the non-hematopoietic environment [42]. The aging non-hematopoietic environment may influence NK cell maturation and function by downregulating the levels of transcription factors Eomes and T-bet. This is supported by the fact that their levels recovered in the young environment [42]. This indicates external factors cause the senescent phenotype, as demonstrated by other studies [42, 68].

#### MicroRNA

MicroRNAs are short (approximately 22 nt), endogenously activated, non-coding RNAs that act as post-transcriptional regulatory factors to negatively regulate gene expression [71]. Each microRNA can regulate multiple mRNAs, and an mRNA can also be regulated by multiple microRNAs [72]. The microRNA expression profile of NK cells changes with aging. For instance, the decreased level of miR-181a-5p leads to developmental defects and decreased function of NK cells [73]. It is suggested that we may preserve or reestablish the function of NK cells in the elderly by adjusting the expression levels of microRNAs.

### NK cells in the tumor microenvironment

#### Impaired NK cell viability and function in the tumor microenvironment

The immunosuppressive tumor microenvironment (TME) impairs NK cell viability and function through multiple mechanisms. Regulatory T cells (Tregs) secrete TGF-β, which inhibits NK cell proliferation and IFN-γ production [74]. Tumor-associated macrophages (TAMs) further dampen NK cell activity through CD80/CD86 signaling [75]. Additionally, tumor-derived PGE2 and TGF-β downregulate activating receptors such as NKp30, NKp44, and NKG2D, thereby reducing NK cell cytotoxicity [76].

Beyond immunosuppressive factors, metabolic stress in the TME significantly disrupts NK cell function. Nutrient deprivation, hypoxia, acidic pH, and metabolic waste accumulation not only affect tumor cells but also severely impair NK cell activity. NK cells rely on both glycolysis and oxidative phosphorylation (OXPHOS) for energy metabolism, and disruption of either pathway reduces IFN-γ secretion and cytotoxic potential [77]. In multiple myeloma, hypoxia suppresses perforin and granzyme B expression while downregulating activating receptors like NKG2D [78]. Collectively, these metabolic disturbances contribute to the functional impairment of NK cells in antitumor immunity.

#### Metabolic reprogramming of NK cells in the tumor microenvironment

Metabolic reprogramming refers to the process by which cells alter their metabolic pathways and energy utilization to adapt to environmental changes or functional demands. Originally described in cancer biology (e.g., the Warburg effect), this concept has since been extended to immune cells, including NK cells, which dynamically modulate their metabolism to regulate effector functions [79]. The metabolic plasticity of NK cells represents a fundamental mechanism for their adaptation to the TME and enhancement of antitumor activity.

Studies indicate that the TME typically suppresses glucose metabolism in NK cells, impairing both glycolysis and OXPHOS, thereby diminishing cytotoxic function. However, research by Ali A. Ashkar and colleagues revealed that NK cells exhibiting a “Warburg-like” metabolic shift—while retaining metabolic flexibility—not only sustain viability under metabolically hostile conditions but also significantly enhance tumor-killing capacity [80]. Hypoxia triggers APOBEC3G-mediated RNA editing to promote stress adaptation [81]. Furthermore, the IRE1α-XBP1-MYC axis contributes to NK cell activation, and SREBP regulates the citrate-malate shuttle system to influence the production of effector molecules such as IFN-γ and granzyme B [82, 83]. These findings not only establish metabolic reprogramming as a critical determinant of NK cell functionality in the TME but also provide a conceptual framework for developing next-generation NK cell-based immunotherapies through metabolic modulation.

## Clinical associations of NK cell immunosenescence

Numerous clinical studies have established that the physiological deterioration of NK cell activity in the elderly serves as a significant risk factor for multiple morbidities, with particularly strong associations observed for infectious diseases, atherosclerosis, and malignant tumors. Additionally, studies have indicated that the mortality rate is higher in the elderly with lower NK cell counts than in those with higher NK cell counts [30]. The existing data appears to corroborate the idea that NK cell phenotype and function change with the disease progression.

### Infectious diseases

After viral infection in older adults, the NK cell pool is highly skewed and unable to replenish naive NK cells due to inefficient NK cell differentiation or viral-specific NK cell cloning and amplification, both of which may impair NK cell function [41]. As mentioned above, there is a correlation between CMV infection and the increased frequency of CD57^+^ NK cells in the elderly [26]. Campos et al. showed that young adults with CMV seropositivity exhibited a substantially greater frequency of CD57^+^ NK cells than CMV-negative young adults, but a similar frequency to that of older adults with CMV positivity [26]. This highlights the importance of accounting for CMV infection when analyzing NK cell immunosenescence and cancer surveillance in the elderly. Abnormal clonal expansion of KIR^+^NKG2C^+^ NK cells occurred in patients with CMV infection, which accounted for more than 50% of the total NK cells [84]. Similarly, multiple studies have shown that NKG2C^bright^ NK cells expand during infections with HIV, hantavirus, hepatitis B virus (HBV), and hepatitis C virus (HCV) [41, 85–87]. The profile of NK cell activation in COVID-19 indicates a correlation between disease severity and CD56^bright^ NK cell arming [88, 89]. Guo et al. found that the aggregation of CD52^+^NKG2C^+^CD94^+^ NK cell subsets was associated with the severity of COVID-19 by single-cell sequencing [39].

### Atherosclerosis

Atherosclerosis is a disease caused by repeated damage to the arterial wall. The risk of developing atherosclerosis and other diseases increases in the elderly, and this is associated with reduced NK cell activity [90]. Research by Guma et al. demonstrated that CMV-induced expansion of NKG2C^+^ NK cells (adaptive NK cells) correlates with the instability of carotid atherosclerotic plaque (CAP). High-risk patients exhibited elevated levels of NKG2C^+^ NK cells compared to lower-risk individuals [91]. However, Alsulami K found the reverse effect, where adaptive NK cell enrichment in CMV-seropositive subjects correlated with decreased coronary plaque burden [92]. This disparity lacks a conclusive explanation. It may be that the protective and pathogenic roles of adaptive NK cells differ at different stages of atherosclerosis. Another possibility is that carotid and coronary atherosclerosis differ in their biological development. To understand this variation in outcomes, further research is required.

### Malignant tumors

NK cells can control the growth of tumor cells during the occurrence and development of malignant tumors. However, the tumor microenvironment induces functional alterations in NK cells that facilitate immune evasion, ultimately promoting malignant growth and metastasis [93]. Degos and colleagues revealed significant alterations in NK cell biology within tumor microenvironments. Their research demonstrated that while tumors exhibit overall diminished NK cell infiltration, the proportion of CD56^bright^ NK cells increases [93]. Notably, the tumor-resident CD103^+^ NK cell subset displayed substantially higher surface expression of co-inhibitory molecules, such as T cell immunoreceptor with Ig and ITIM domains (TIGIT) and T cell immunoglobulin and mucin domain-containing protein 3 (Tim-3), compared to CD103^−^ NK cells. Most importantly, these intratumoral NK cells manifested functional impairment through markedly reduced granzyme B (GZMB) production when contrasted with NK cells from neighboring healthy tissues, suggesting substantial suppression of their cytotoxic capacity in the tumor milieu [93]. Furthermore, tumors exhibited decreased levels of the cytotoxic cytokine IL-15 and elevated levels of IL-6, which inhibits the STAT-5 pathway and NK cell activity [94]. When compared to nearby NK cells in healthy tissue, tumor-resident NK cells produced less GZMB, indicating a potential loss of cytotoxic activity [93].

In lung cancer, the downregulation of NKp30 and NKG2D results in an inhibitory phenotype for NK cells, and a reduction in GZMB impairs their cytolytic function [95, 96]. In breast tumors, the expression of NK cell activating receptors is decreased, whereas the expression of inhibitory receptors is increased, limiting the antitumor immunity of NK cells [97]. The research showed that the endometrial cancer tumor microenvironment has a significant impact on resident NK cells, as it can decrease their cytotoxic capacity and remodel their phenotype and function, ultimately promoting tumor progression [93]. Chronic myeloid leukemia (CML) is a disease associated with aging (about half of all cases are diagnosed in people over 65 years of age) [98]. Some studies have revealed that CML patients experience progressive functional deterioration of NK cells at all stages of the disease [98]. Patients with CML exhibit higher levels of CD57 expression in the bone marrow and TIGIT expression on the surface of NK cells in peripheral blood. Because decreased proliferation is linked to high expression of CD57, there is a decrease in NK cell proliferation in the bone marrow [99]. NK cells from patients with acute myeloid leukemia (AML) also downregulate the expression of activating receptors NKp30, NKp46, and DNAM-1 [100, 101].

Notably, NK cell senescence appears to be not merely an immunological disorder but may also constitute an adaptive mechanism preserved through evolution for host life cycle regulation. Uterine natural killer (uNK) cells are abundantly present at the maternal-fetal interface [102]. During early pregnancy, soluble HLA-G secreted by fetal trophoblasts binds to CD158d of uNK cells, activating the DNA damage response pathway and promoting a senescent phenotype [103, 104]. This process generates various SASP factors that facilitate angiogenesis and trophoblast invasion [103]. This reveals the potential physiological significance of NK cellular senescence during pregnancy, though this mechanism remains to be further validated by additional research.

## Autologous NK cell infusion therapy: rejuvenating immune function

By expanding and reinfusing autologous NK cells in vitro, this therapy can effectively clear senescent cells, improve immune function, and mitigate systemic inflammatory responses. A randomized controlled trial involving 37 healthy middle-aged volunteers found that subjects receiving autologous NK cell infusions exhibited a significant reduction in senescent and exhausted T cell subsets (CD28^−^ and CD57^+^ subsets), along with decreased expression of immunosuppressive markers such as PD-1^+^ and TIM-3^+^ [105]. Furthermore, levels of key pro-inflammatory cytokines (IL-6, IL-8, IL-17) were markedly reduced, suggesting that NK cell therapy alleviates age-related “inflammaging“ [105]. Additional research revealed that expanded NK cells selectively eliminate senescent CD4^+^ T cells while sparing normal CD4^+^ T cells, a specificity potentially mediated by altered surface receptor expression (e.g., downregulation of NKG2C/KLRG1 and upregulation of NKG2A/TIM3) [105]. In aged mouse models, the combination of autologous NK cells with dopamine-releasing peptides (opioid peptides) synergistically enhanced the clearance efficiency of senescent cells, outperforming monotherapy while improving tissue function and extending healthspan [106]. Another small-scale trial involving five healthy elderly individuals demonstrated that in vitro-activated and reinfused NK cells reduced the levels of senescence markers (p16, β-galactosidase), with effects lasting over eight months and significantly decreasing inflammatory markers [107]. These findings highlight the potential of autologous NK cell therapy in mitigating immunosenescence and suggest its broader role in delaying overall aging.

Currently, NK cell therapy is emerging as an innovative anti-senescence approach, not only capable of restoring age-compromised immune function but also offering new avenues for treating various age-related diseases. However, translating these findings into clinical applications requires further exploration of optimal treatment timing, dosage, and the efficacy and safety of NK cells from different sources. With advancing research, we may witness the rise of a novel medical strategy—leveraging NK cell therapy to help humanity better combat aging and its associated challenges.

## Conclusions

Immunosenescence, the age-related decline of immune function, plays a crucial role in both the normal aging processes and the development of various age-related diseases. As key effectors of innate immunity, NK cells exhibit distinct phenotypic and functional alterations during senescence that significantly compromise overall immune competence. The most well-established biomarker of NK cell aging is the progressive accumulation of CD57^+^ cells, which indicates terminal differentiation and reduced proliferative capacity. Concurrently, aging induces a characteristic shift in NK cell subsets, characterized by the expansion of the cytotoxic CD56^dim^CD16^+^ population and the contraction of the immunoregulatory CD56^bright^CD16^−^ subset. These changes are accompanied by significant alterations in receptor expression patterns and cytokine production profiles, ultimately leading to functional impairments through multiple mechanisms, including diminished cytotoxic activity, reduced interferon-γ secretion, and impaired responsiveness to cytokine stimulation. The clinical implications of NK cell senescence are particularly evident in malignancies, chronic viral infections, and autoimmune disorders, where the senescent phenotype is associated with worse clinical outcomes. This understanding has sparked increasing interest in developing innovative NK cell-based therapeutic strategies to counteract the effects of immunosenescence, such as ex vivo expansion and reinfusion of autologous NK cells, genetic engineering approaches to enhance cytotoxic potential, and combination therapies with immunomodulatory agents. Future research should focus on elucidating the molecular mechanisms underlying NK cell senescence, identifying more precise biomarkers for clinical monitoring, and developing targeted interventions to either reverse the senescent phenotype or harness its unique features for therapeutic benefit. As global demographics continue to shift toward an aging population, enhancing our understanding of NK cell immunosenescence will be essential for developing effective treatments and improving clinical outcomes in age-related diseases, ultimately contributing to healthier aging.

## Data Availability

No datasets were generated or analysed during the current study.

## Abbreviations

ADCC: Antibody-dependent cell-mediated cytotoxicity
AGEs: Advanced glycation end products
AML: Acute myeloid leukemia
CAP: Carotid atherosclerotic plaque
CMV: Cytomegalovirus
CML: Chronic myeloid leukemia
cNK: conventional NK
COVID-19: Coronavirus disease 2019
DDR: DNA damage response
EndoG: endonuclease G
Eomes: Eomesodermin
GO: Glyoxal
GPCR21: G protein-coupled receptor 21
GZMB: Granzyme B
HBV: Hepatitis B virus
HCV: Hepatitis C virus
HIV: Human immunodeficiency virus
iNK: immature NK
MGO: Methylglyoxal
mNK: mature NK
MRCC: Metastatic renal cell carcinoma
NK: Natural killer
OXPHOS: Oxidative phosphorylation
PGC-1α: PPAR-gamma coactivator 1-alpha
Pls: Plasmalogens
ROS: Reactive oxygen species
STAT5: Signal transducer and activator of transcription 5
TAMs: Tumor-associated macrophages
TERT: Telomerase reverse transcriptase
TIGIT: T cell immunoreceptor with Ig and ITIM domains
Tim-3: T cell immunoglobulin and mucin domain-containing protein 3
TME: Tumor microenvironment
Treg cells: Regulatory T cells
uNK: Uterine natural killer
